# Evaluation of Nutritional Support and In-Hospital Mortality in Patients With Malnutrition

**DOI:** 10.1001/jamanetworkopen.2020.33433

**Published:** 2021-01-20

**Authors:** Nina Kaegi-Braun, Marlena Mueller, Philipp Schuetz, Beat Mueller, Alexander Kutz

**Affiliations:** 1 Division of Endocrinology, Diabetes, and Metabolism, University Department of Medicine, Kantonsspital Aarau, Aarau, Switzerland; 2 Division of General Internal and Emergency Medicine, University Department of Medicine, Kantonsspital Aarau, Aarau, Switzerland; 3 Faculty of Medicine, University of Basel, Basel, Switzerland

## Abstract

**Importance:**

Malnutrition affects a considerable proportion of patients in the hospital and is associated with adverse clinical outcomes. Recent trials found a survival benefit among patients receiving nutritional support.

**Objective:**

To investigate whether there is an association of nutritional support with in-hospital mortality in routine clinical practice.

**Design, Setting, and Participants:**

This cohort study was conducted from April 2013 to December 2018 among a population of patients from Swiss administrative claims data. From 114 264 hospitalizations of medical patients with malnutrition, 34 967 patients (30.6%) receiving nutritional support were 1:1 propensity score matched to patients with malnutrition in the hospital who were not receiving nutritional support. Patients in intensive care units were excluded. Data were analyzed from February 2020 to November 2020.

**Exposures:**

Receiving nutritional support, including dietary advice, oral nutritional supplementation, or enteral and parenteral nutrition.

**Main Outcomes and Measures:**

The primary outcome was all-cause in-hospital mortality. Secondary outcomes were 30-day all-cause hospital readmission and discharge to a postacute care facility. Poisson and logistic regressions were used to estimate incidence rate ratios (IRRs) and odds ratios (ORs) of outcomes.

**Results:**

After matching, the study identified 69 934 hospitalizations of patients coded as having malnutrition in the cohort (mean [SD] age, 73.8 [14.5] years; 36 776 [52.6%] women). Patients receiving nutritional support, compared with those not receiving nutritional support, had a lower in-hospital mortality rate (2525 of 34 967 patients died [7.2%] vs 3072 of 34 967 patients died [8.8%]; IRR, 0.79 [95% CI, 0.75-0.84]; *P* < .001) and a reduced 30-day readmission rate (IRR, 0.95 [95% CI, 0.91-0.98]; *P* = .002). In addition, patients receiving nutritional support were less frequently discharged to a postacute care facility (13 691 patients [42.2%] vs 14 324 patients [44.9%]; OR, 0.89 [95% CI, 0.86-0.91]; *P* < .001).

**Conclusions and Relevance:**

These findings suggest that nutritional support was associated with reduced mortality among patients in the medical ward with malnutrition. The results support data found by randomized clinical trials and may help to inform patients, clinicians, and authorities regarding the usefulness of nutritional support in clinical practice.

## Key Points

**Question:**

Is nutritional support as prescribed in clinical practice associated with a mortality benefit among patients with malnutrition?

**Findings:**

In this cohort study of 69 934 patients with malnutrition in a nationwide Swiss claims database, the in-hospital mortality rate was significantly lower among patients receiving nutritional support compared with those not receiving nutritional support.

**Meaning:**

This study found that nutritional support was associated with a mortality benefit, highlighting the importance of nutritional support for patients in the hospital with malnutrition.

## Teaser

This cohort study examines the association of nutritional support with mortality rates among hospitalized patients with malnutrition in Switzerland.

## Introduction

Malnutrition is defined as a state of insufficient intake or uptake of nutrients, leading to an altered body composition.^1^ As a risk factor associated with adverse outcomes, it is diagnosed in a considerable proportion of patients in hospitals.^2,3^ Among patients in hospitals in Switzerland, a 2010 study^4^ found a malnutrition prevalence rate of 18.2% and a 2015 study^2^ found a rate of 27.8%, using the nutritional risk screening (NRS 2002) score.^5^ Patients who are older and considered frail have high rates of illness-related impaired protein and energy homeostasis, hormonal changes, and reduction of appetite associated with disease-related malnutrition.^1,6^ Importantly, the association between illness and nutritional status seems to be bidirectional, with malnutrition also associated with morbidity, mortality, functional decline, prolonged hospital stays, and higher health care costs.^2,7^

In light of the high number of patients with malnutrition in the hospital, awareness of the detrimental associations of malnutrition among patients with multiple conditions has increased.^8,9^ While a 2016 study^10^ did not find an association between nutritional interventions and mortality or other clinical outcomes, randomized clinical trials from 2019^11^ and 2016^12^ found that nutritional support reduced risks for mortality and complications and improved functional outcomes and quality of care. Therefore, current practice guidelines issued by the European Society for Clinical Nutrition and Metabolism^3^ and the American Society for Parenteral and Enteral Nutrition^13^ suggest screening for malnutrition, nutritional assessment, and nutritional support for inpatients with malnutrition.

An increasing number of hospitals are implementing strategies for more standardized screening and management of patients with malnutrition. As such, we aimed to use a large Swiss nationwide claims database to assess whether the administration of nutritional support in clinical routine was associated with improved in-hospital survival compared with receiving no nutritional support.

## Methods

### Study Design

The institutional review board of Northwestern Switzerland approved this cohort study and waived the requirement of participant informed consent, as the data were deidentified. This study followed the Strengthening the Reporting of Observational Studies in Epidemiology (STROBE) reporting guideline.

We conducted a nationwide population-based cohort study using an administrative claims database (ie, Medizinstatistik) provided by the Federal Statistical Office in Switzerland. The database contains longitudinal, individual-level data on in-hospital health care use, inpatient diagnoses, diagnostic tests, and procedures. It includes all Swiss inpatient discharge records from acute care, general, and specialty hospitals, excluding hospital units of postacute care institutions, regardless of payer. The database thus provides a near-complete sample of hospitalizations in Switzerland. Each hospitalization is identified uniquely in the database, so rehospitalizations could be tracked. As data are assessed for every hospital stay, a single patient may have more than 1 index admission in our cohort.^14^

All covariates were collected for each patient during that patient’s index hospitalization. We also assessed database records for diagnosis and treatment data on the basis of *International Statistical Classification of Diseases and Related Health Problems, Tenth Revision (ICD-10)*^15^ and Swiss Classification of Operations (CHOP) codes, as well as other variables, such as demographic characteristics and admission details (eg, insurance status, month and year of admission, location before admission [eg, home or nursing home], and Swiss hospital teaching level [ie, A,B, or C, in which A is a university hospital or larger and C is a small hospital]). We also calculated factors associated with general health status (ie, Charlson Comorbidity Index, malnutrition severity, hospital frailty risk score,^16^ and use of palliative care) and health care use (ie, length of stay [LOS] in the hospital and number of previous hospitalizations) by means of variables from the database. These covariates were selected a priori on the basis of clinical experience, expertise, and published literature.^17^

### Data Sources and Study Population

We included hospitalizations of adult (age ≥18 years) patients with malnutrition and noncritical illness in medical wards. Hospitalizations with any intensive care unit (ICU) admission were excluded to increase specificity. Medical diagnoses were coded using the *ICD-10, German Modification* (*ICD-10-GM*) codes.^18^ Main diagnoses consisted of the main reason for hospitalization and in-hospital care; secondary diagnoses were a measure for the level of comorbidity. Hospitalizations of patients with a diagnosis of malnutrition were identified using *ICD-10-GM* codes E43, for unspecified severe protein-energy malnutrition; E44, for protein-energy malnutrition of moderate or mild degree; and E46, for unspecified protein-energy malnutrition (eTable 1 in the Supplement). The following codes were not considered, as they are not common reasons for malnutrition in Swiss hospitals: E40, for kwashiorkor; E41, for nutritional marasmus; E42, for marasmic kwashiorkor; and E45, for retarded development following protein-energy malnutrition.

**Figure 1.**
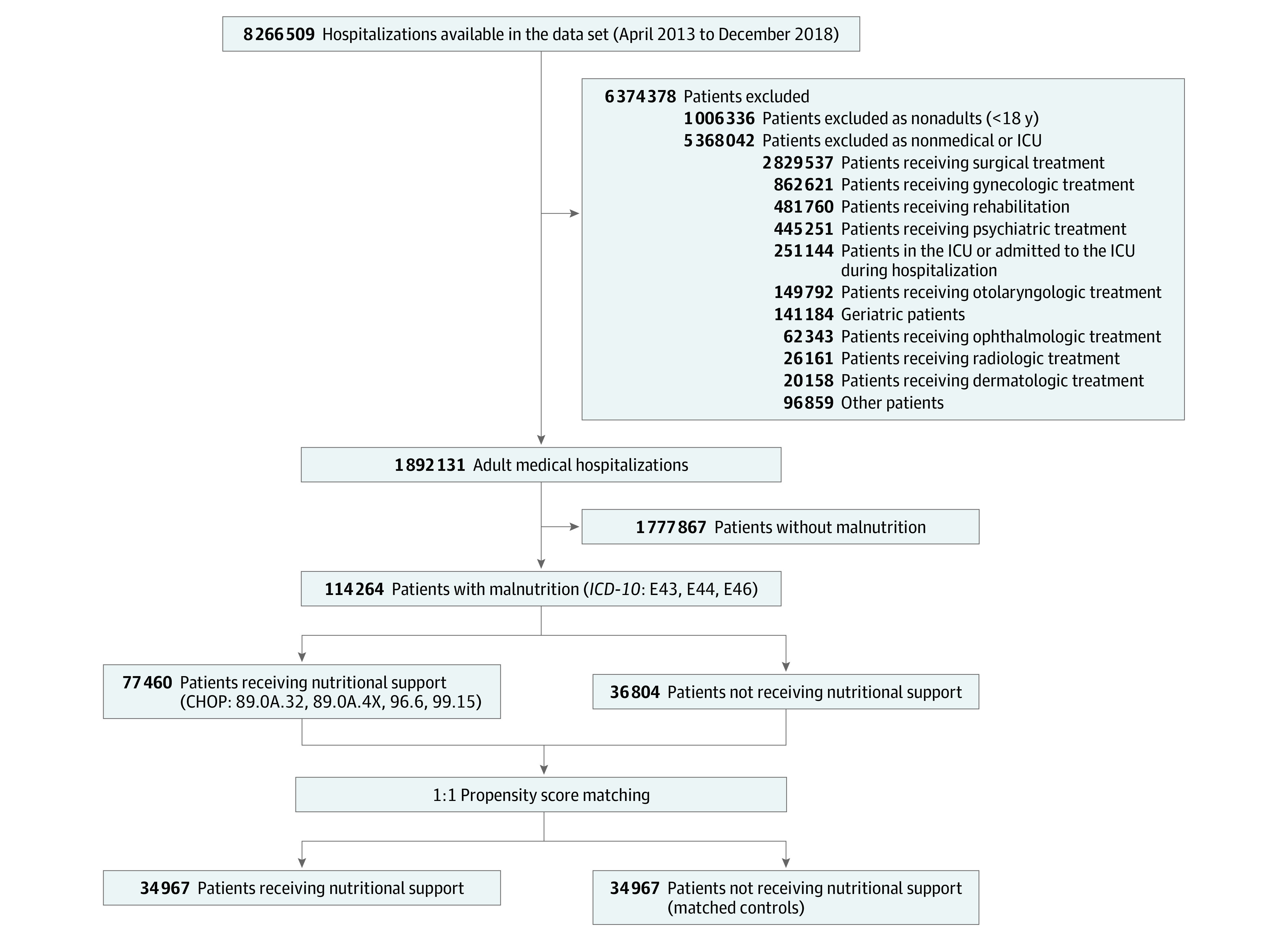
Study Flowchart ICU indicates intensive care unit; *ICD-10*, *International Statistical Classification of Diseases and Related Health Problems, Tenth Revision*; CHOP, Swiss Classification of Operations code.

The study period was from April 2013 to December 2018. In 2012, the Swiss Diagnosis-Related Groups (SwissDRG) reimbursement system was implemented, and since then, coding for malnutrition and nutritional support has gained financial relevance, as reimbursement for hospitalizations with codes for malnutrition is now higher than those without such codes. While encoding rules for malnutrition were relevantly adapted by SwissDRG in March 2013 by basing the *ICD-10* coding mainly on the criteria of the NRS 2002,^19^ only minor changes in coding have been made since then.^20^ The NRS-2002 score assesses the risk for malnutrition and differentiates between patients at nutritional risk (ie, those with ≥3 points) and patients with malnutrition (ie, those with ≥4 points). In this study, we used the term *malnutrition* for all patients with an NRS score of 3 or greater, in accordance with a 2019 clinical trial.^11^ From a total of 1 892 131 adults who were hospitalized during our study period, we identified 114 264 patients (6%) who were eligible based on inclusion and exclusion criteria (Figure 1).

### Exposure

To assess the associations of nutritional support with clinical outcomes of interest, all hospitalizations of patients with malnutrition were screened for the presence of nutritional support based on CHOP codes. The following CHOP codes were counted as nutritional support: 89.0A.32 and 89.0A.4X, for dietary advice and nutritional therapy; 96.6, for enteral infusion of concentrated nutrients; and 99.15, for parenteral infusion of concentrated nutrient solutions.

### Outcomes

The primary outcome was all-cause in-hospital mortality. Secondary outcomes were all-cause 30-day readmission rate and discharge to a postacute care facility. For disposition status, the event of interest was discharge to a short-term or long-term postacute care facility or discharge home.

### Statistical Analysis

To control for confounding by indication, we fitted different Poisson regression models for in-hospital all-cause mortality and 30-day readmission, and results were reported as incidence rate ratios (IRRs) with their respective 95% CIs. Similarly, we reported odds ratios (ORs) for discharge to a postacute care facility using logistic regression models. Comparing hospitalizations of patients receiving nutritional support with hospitalizations of patients not receiving nutritional support, we performed unadjusted and multivariable regression analyses, adjusting for sociodemographic factors (ie, age, sex, nationality, insurance status, month and year of admission, mode of admission, location before admission, hospital size, and hospital site). The fully adjusted model additionally included main diagnoses, comorbidities, severity of malnutrition, total number of hospitalizations, use of palliative treatment, Charlson Comorbidity Index, hospital frailty risk score, and hospital LOS.

We performed a propensity score–matched analysis. Eligible hospitalizations of patients receiving nutritional support were 1:1 propensity score matched to a comparative general medical cohort of patients in the hospital with malnutrition not receiving nutritional support (ie, the matched control population). The probability of receiving nutritional support vs not receiving nutritional support was calculated via a multivariable logistic regression model that contained all baseline covariates and month of admission. No data were missing in our study. The estimated propensity score was used to match hospitalizations of patients with malnutrition receiving nutritional support with patients in a nearest-neighbor control group using a caliper size of 0.0005 on the propensity scale. Covariate balance before and after propensity score matching was assessed using standardized differences. A standardized difference less than 10% was interpreted as indicating adequate balance between groups.^21^

After propensity score matching, estimates of the effect sizes and corresponding 95% CIs were determined using Poisson or logistic regressions as appropriate; the models were additionally adjusted for hospital site. Kaplan-Meier curve was used to illustrate differences in time to in-hospital mortality, and Cox proportional hazards regression was used for the calculation of hazard ratios (HRs).

We performed sensitivity analyses of patients who were treated with only dietary advice or oral nutritional support (ie, excluding patients receiving enteral or parenteral nutrition). For exposed and unexposed groups, we calculated a separate propensity score (using a caliper size of 0.0005) and performed a separate 1:1 matching.

Because in-hospital mortality functions as competing risk associated with the 2 secondary outcomes, we analyzed competing event data using the cumulative incidence function and performed the nonparametric Gray test to compare these functions.^22^ To explore the consistency of the findings among subgroups, we included interaction terms in the regression models to test for modification associated with baseline factors. Specifically, we tested for subgroups by patient age, sex, main diagnosis during hospitalization, comorbidities, degree of malnutrition, hospital frailty risk score, and hospital LOS.

All *P* values were 2-sided and were not adjusted for multiple testing, and *P* < .05 was considered statistically significant. All statistical analyses were performed using Stata statistical software version 15.1 (StataCorp). Data were analyzed from February 2020 to May 2020.

## Results

Among 69 934 patients with malnutrition who were hospitalized and propensity score matched, the mean (SD) age was 73.8 (14.5) years, 36 776 (52.6%) were women, 53 849 patients (77.0%) were admitted to a tertiary care hospital, 30 933 patients (44.2%) had hypertension, 23 894 patients (34.2%) had cancer, 22 598 patients (32.3%) had chronic kidney failure, and 7662 patients (10.9%) had severe malnutrition. Most patients had multiple hospital admissions and a high level of multimorbidity (Table 1).

**Table 1.** Baseline Patient Characteristics

Characteristic	Before matching				After matching			
	No. (%)		*P* value	Standardized difference	No. (%)		*P* value	Standardized difference
	No nutritional support (n = 36 804)	Nutritional support (n = 77 460)			No nutritional support (n = 34 967)	Nutritional support (n = 34 967)		
Age, mean (SD)	74.1 (14.8)	72.7 (14.7)	<.001	0.091	73.8 (14.9)	73.7 (14.1)	.33	0.007
Women	19 460 (52.9)	40 611 (52.4)	.16	0.009	18 430 (52.7)	18 346 (52.5)	.52	0.005
Swiss residency	31 392 (85.3)	67 759 (87.5)	<.001	0.064	29 983 (85.7)	29 914 (85.5)	.46	−0.006
Public insurance	28 782 (78.2)	60 104 (77.6)	.02	−0.015	27 338 (78.2)	27 346 (78.2)	.94	0.001
Admission data								
Emergency admission	27 168 (73.8)	58 129 (75.0)	<.001	−0.028	26 036 (74.5)	25 958 (74.2)	.50	0.005
Admission from home	29 284 (79.6)	64 367 (83.1)	<.001	−0.091	28 196 (80.6)	28 225 (80.7)	.78	−0.002
Tertiary hospital	27 898 (75.8)	63 361 (81.8)	<.001	−0.147	27 032 (77.3)	26 817 (76.7)	.05	0.015
Main diagnosis								
Endocrine condition	1890 (5.1)	4210 (5.4)	.04	−0.013	1803 (5.2)	1838 (5.3)	.55	−0.005
Cardiac condition	4445 (12.1)	8509 (11.0)	<.001	0.034	4246 (12.1)	4232 (12.1)	.87	0.001
Infection	1860 (5.1)	3956 (5.1)	.70	−0.002	1806 (5.2)	1755 (5.0)	.38	0.007
Respiratory condition	5831 (15.8)	9818 (12.7)	<.001	0.091	5261 (15.0)	5294 (15.1)	.73	−0.003
Cancer	8336 (22.6)	21 525 (27.8)	<.001	−0.119	8112 (23.2)	8203 (23.5)	.42	−0.006
Psychiatric condition	1826 (5.0)	2969 (3.8)	<.001	0.055	1674 (4.8)	1681 (4.8)	.90	−0.001
Neurological condition	1079 (2.9)	2460 (3.2)	.03	−0.014	1059 (3.0)	1021 (2.9)	.40	0.006
Gastrointestinal condition	3205 (8.7)	8166 (10.5)	<.001	−0.062	3157 (9.0)	3132 (9.0)	.74	0.003
Musculoskeletal condition	1378 (3.7)	2801 (3.6)	.28	0.007	1315 (3.8)	1342 (3.8)	.59	−0.004
Kidney condition	1619 (4.4)	3074 (4.0)	<.001	0.022	1542 (4.4)	1528 (4.4)	.80	0.002
Comorbidities								
Diabetes	6062 (16.5)	13 370 (17.3)	<.001	−0.021	5860 (16.8)	5878 (16.8)	.86	−0.001
Coronary heart disease	5656 (15.4)	12 401 (16.0)	.005	−0.018	5470 (15.6)	5398 (15.4)	.45	0.006
Hypertension	16067 (43.7)	35 002 (45.2)	<.001	−0.031	15 484 (44.3)	15 449 (44.2)	.79	0.002
Liver disease	2554 (6.9)	4982 (6.4)	.001	0.020	2412 (6.9)	2470 (7.1)	.39	−0.007
Cancer	12 254 (33.3)	30 501 (39.4)	<.001	−0.127	11 895 (34.0)	11 999 (34.3)	.41	−0.006
Chronic kidney failure	11 847 (32.2)	24 750 (32.0)	.42	0.005	11 327 (32.4)	11 271 (32.2)	.65	0.003
COPD	6187 (16.8)	11 617 (15.0)	<.001	0.050	5684 (16.3)	5726 (16.4)	.67	−0.003
Heart failure	8640 (23.5)	17 910 (23.1)	.19	0.008	8299 (23.7)	8243 (23.6)	.62	0.004
Pneumonia	5585 (15.2)	11 262 (14.5)	.005	0.018	5246 (15.0)	5256 (15.0)	.92	−0.001
General health–associated factors								
Severe malnutrition	3793 (10.3)	25 325 (32.7)	<.001	−0.566	3793 (10.8)	3869 (11.1)	.36	−0.007
Receiving palliative treatment	1841 (5.0)	4869 (6.3)	<.001	−0.056	1777 (5.1)	1835 (5.2)	.32	−0.007
CCI score, mean (SD)	3.55 (3.3)	3.85 (3.4)	<.001	−0.092	3.60 (3.3)	3.61 (3.3)	.52	−0.005
Hospital frailty risk score								
<5	18 851 (51.2)	39 456 (50.9)	<.001	0.005	17 903 (51.2)	17 858 (51.1)	<.001	0.010
5-15	15 536 (42.2)	33 362 (43.1)			14 763 (42.2)	15 064 (43.1)		
>15	2417 (6.6)	4642 (6.0)			2301 (6.6)	2045 (5.8)		
Health care use								
Hospital LOS, median (IQR), d	9 (6-15)	12 (8-19)	<.001	−0.287	10 (6-16)	10 (7-15)	<.001	−0.021
Hospitalizations, No.								
1	2819 (7.7)	6615 (8.5)	<.001	0.072	2745 (7.9)	2646 (7.6)	.06	0.003
2-5	12 613 (34.3)	28 762 (37.1)			12 115 (34.6)	12 389 (35.4)		
>5	21 372 (58.1)	42 083 (54.3)			20 107 (57.5)	19 932 (57.0)		

Abbreviations: CCI, Charlson Comorbidity Index; COPD, Chronic obstructive pulmonary disease; IQR, interquartile range; LOS, length of stay.

Even before propensity score matching, nearly all baseline characteristics were well balanced, with a few exceptions. Patients receiving nutritional support, compared with those not receiving nutritional support, had a lower mean [SD] age (72.7 [14.7] years vs 74.1 [14.8] years), higher prevalence of severe malnutrition (25 325 patients [32.7%] vs 3793 patients [10.3%]), higher prevalence of oncology admissions (21 525 patients [27.8%] vs 8336 patients [22.6%]) and cancer as comorbidity (30 501 patients [39.4%] vs 12 254 patients [33.3%]), and longer median (interquartile range) hospital LOS (12 [8-19] days vs 9 [6-15] days] (Table 1). After propensity score matching all baseline characteristics were well balanced with a standardized difference below 10%. Among all patients after matching, 3426 patients (4.9%) received enteral nutrition and 1629 patients (2.4%) received parenteral nutrition.

### Primary Outcome

Among 77 460 patients who were unmatched and receiving nutritional support, 6633 patients (8.6%) died during hospitalization, compared with 3239 patients (8.8%) who died among 36 804 patients who were unmatched and not receiving nutritional support. This outcome corresponded to an unadjusted IRR of 0.77 (95% CI, 0.74-0.80; *P* < .001) for in-hospital mortality associated with receiving nutritional support vs not receiving nutritional support. We also found that nutritional support was significantly associated with reduced mortality rate in the fully adjusted model, with an IRR of 0.76 (95% CI, 0.73-0.80; P <.001).

**Figure 2.**
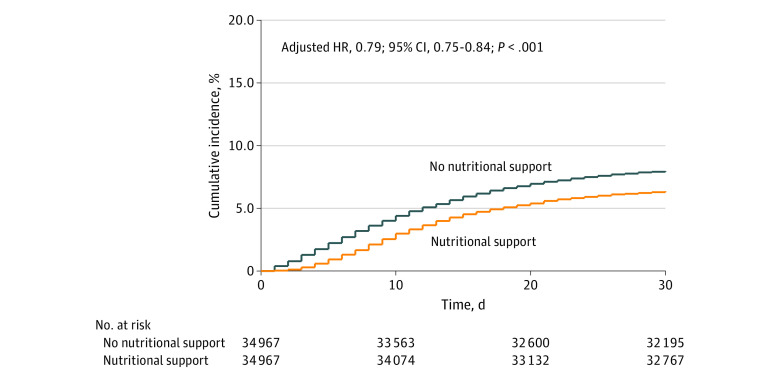
Kaplan-Meier Curve for Propensity Score–Matched Rate of All-Cause In-Hospital Mortality HR indicates hazard ratio. HR was adjusted by hospital site.

Among 69 934 patients who were propensity score matched, 5597 patients died, for an overall all-cause in-hospital mortality rate of 8.0%. Among matched patients, 2525 of 34 967 patients receiving nutritional support died, for a mortality rate of 7.2%, compared with 3072 of 34 967 patients who died in the matched control group, for a mortality rate of 8.8% (IRR, 0.79 [95% CI, 0.75-0.84]; *P* < .001) (Table 2). Kaplan-Meier survival curve showed lower cumulative incidence of in-hospital deaths over 30 days for the group receiving nutritional support, with an adjusted HR of 0.79 (95% CI, 0.75-0.84; *P* < .001) (Figure 2).

**Table 2.** Association of Nutritional Therapy With Clinical Outcomes Before and After Matching

Outcome	No nutritional support	Nutritional support	*P* value
**In-hospital all-cause mortality**			
Patients, No.	36 804	77 460	NA
Events, No. (%)	3239 (8.8)	6633 (8.6)	NA
IRR (95% CI)			
Unadjusted	1 [Reference]	0.77 (0.74-0.80)	<.001
Adjusted^a^	1 [Reference]	0.76 (0.73-0.80)	<.001
Fully adjusted^b^	1 [Reference]	0.76 (0.73-0.79)	<.001
After propensity score matching^c^			
Patients, No.	34 967	34 967	NA
Events, No. (%)	3072 (8.8)	2525 (7.2)	NA
IRR (95% CI)	1 [Reference]	0.79 (0.75-0.84)	<.001
**30-d readmission rate**			
Patients, No.	33 565	70 827	NA
Events, No. (%)	6490 (19.3)	13 010 (18.4)	NA
IRR (95% CI)			NA
Unadjusted	1 [Reference]	0.94 (0.91-0.97)	<.001
Adjusted^a^	1 [Reference]	0.95 (0.92-0.98)	.001
Fully adjusted^b^	1 [Reference]	0.95 (0.92-0.98)	.003
After propensity score matching^c^			
Patients, No.	31 895	32 442	NA
Events, No. (%)	6077 (19.1)	5950 (18.3)	NA
IRR (95% CI)	1 [Reference]	0.95 (0.91-0.98)	.002
**Discharge to postacute care facility**			
Patients, No.	33 565	70 827	NA
Events, No. (%)	15 223 (45.4)	31 166 (44.0)	NA
OR (95% CI)			
Unadjusted	1 [Reference]	0.95 (0.92-0.97)	<.001
Adjusted^a^	1 [Reference]	1.04 (1.01-1.07)	.008
Fully adjusted^b^	1 [Reference]	0.95 (0.92-0.98)	<.001
After propensity score matching^c^			
Patients, No.	31 895	32 442	NA
Events, No. (%)	14 324 (44.9)	13 691 (42.2)	NA
OR (95% CI)	1 [Reference]	0.89 (0.86-0.91)	<.001

Abbreviations: IRR, incidence rate ratio; NA, not applicable; OR, odds ratio.

^a^ Adjusted for sociodemographic factors: age, sex, nationality, insurance status, month and year of admission, mode of admission, location before admission, hospital size, and hospital site.

^b^ Adjusted for sociodemographic factors and medical factors: main diagnosis, comorbidities, severity of malnutrition, total number of hospitalizations, use of palliative treatment, Charlson Comorbidity Index, hospital frailty risk score, and hospital length of stay.

^c^ Sociodemographic factors and medical factors used in propensity score matching and analyses adjusted for hospital site.

### Secondary Outcomes

In the unadjusted analysis, we found significantly lower 30-day readmission rates among patients receiving nutritional support (IRR, 0.94 [95% CI, 0.91 to 0.97]; P < .001) compared with patients not receiving nutritional support. Similarly, risk of 30-day readmission was lower in the Poisson model adjusted for sociodemographic characteristics (IRR, 0.95 [95% CI, 0.92-0.98]; *P* = .001) and the fully adjusted model (IRR, 0.95 [95% CI, 0.92-0.98]; *P* = .003). After propensity score matching, there was still a significant difference in 30-day readmission rate between the group receiving nutritional support compared with the matched reference group (5950 of 32 442 patients [18.3%] vs 6077 of 31 895 patients [19.1%]; IRR, 0.95 [95% CI, 0.91-0.98]; *P* = .002).

The results for admission to a postacute care institution were heterogenous in the nonmatched population, while after propensity score matching, patients receiving nutritional support were significantly less frequently admitted to a postacute care facility compared with matched patients in the reference group (13 691 patients [42.2%] vs 14 324 patients [44.9%]; OR, 0.89 [95% CI, 0.86-0.91]; *P* < .001) (Table 2). Using the Gray method to account for competing risk after matching, nutritional support was also associated with a lower 30-day readmission rate (HR, 0.95 [95% CI, 0.92-0.98]; *P* =.001), while there was no risk reduction associated with discharge to a postacute facility (HR, 1.05 [95% CI, 1.02-1.07]; *P* < .001).

#### Sensitivity and Subgroup Analyses

**Figure 3.**
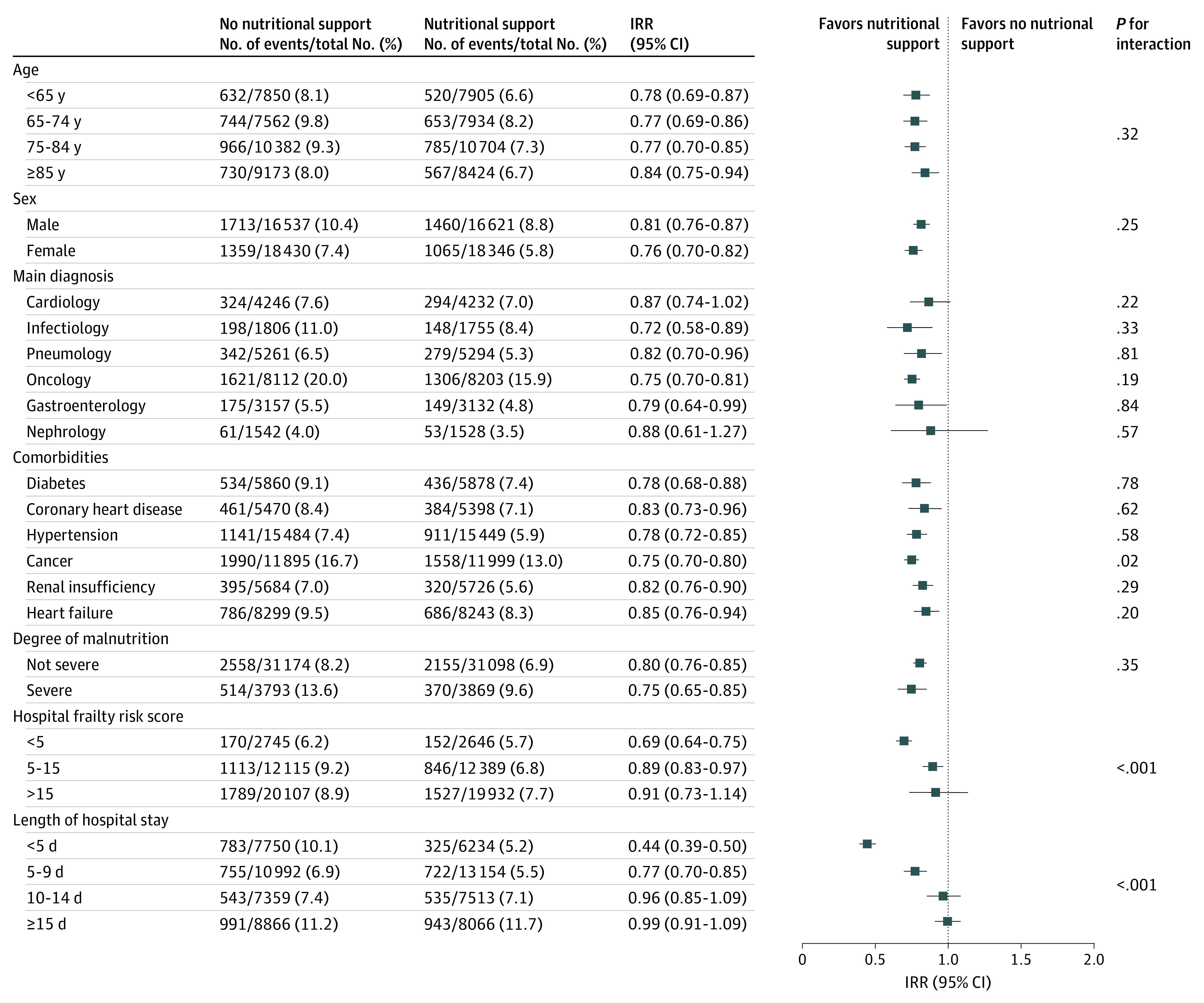
Propensity Score–Matched Incidence Rate Ratios (IRRs) for In-Hospital Mortality Stratified by Patient Characteristic Stratified by age, sex, main diagnoses, comorbidities, degree of malnutrition, hospital frailty risk score, and length of hospital stay.

Among patients receiving nutritional support only (ie, when excluding patients receiving enteral or parenteral nutrition), after matching, the IRR for overall mortality was 0.75 (95% CI, 0.71-.080; *P* < .001) compared with patients not receiving nutritional support (eTable 2 in the Supplement). Using the propensity score–matched population, we performed several subgroup analyses stratifying for main baseline covariates (Figure 3). In general, our findings for in-hospital mortality remained robust, except for the following conditions: among patients who were hospitalized with a lower hospital frailty risk score (ie, <5 points), receiving nutritional support was associated with a greater reduction in mortality risk compared with patients with higher frailty risk scores. Among patients with a shorter hospital LOS (ie, <5 days), prescribed nutritional intervention was associated with a greater decrease in mortality risk compared with those who were hospitalized more than 5 days.

## Discussion

This large population-based cohort study of more than 110 000 patients with malnutrition in hospitals had 2 key findings. First, we observed a lower rate of in-hospital mortality among patients with malnutrition receiving nutritional support compared with those not receiving nutritional support. The magnitude of 21% relative risk reduction was high and comparable to findings from randomized clinical trials from 2019^11^ and 2016^12^ and a 2019 meta-analysis.^23^ Second, our subgroup analyses suggested that patients with lower frailty risk scores and patients with shorter hospital stays may have a larger benefit from nutritional support as performed in daily clinical routine.

Although we do not have postdischarge mortality data in our data set, our main finding is in line with findings from previous randomized clinical trials. The largest multicenter Effect of Early Nutritional Support on Frailty, Functional Outcomes, and Recovery of Malnourished Medical Inpatients Trial (EFFORT)^11^ completed so far, to our knowledge, included 2028 patients and reported a 35% risk reduction of 30-day mortality in patients randomized to individualized nutritional support during hospital stay. The Nutrition Effect on Unplanned Readmissions and Survival in Hospitalized Patients (NOURISH) study^12^ evaluated the effects of adding a specialized nutrient-dense oral nutritional supplement compared with placebo supplement among patients with malnutrition who were in the hospital for respiratory or cardiac care. This trial showed a 51% reduction in 90-day-overall-mortality among treated patients. An updated meta-analysis^23^ using individualized patient data reported a 27% relative risk reduction in overall mortality up to 6 months after discharge.

Moreover, our cohort of patients with malnutrition who were not selected differed from those in the clinical studies mentioned above because it included a higher proportion of patients receiving enteral nutrition (4.9% after matching) or parenteral nutrition (2.4% after matching). For example, the EFFORT trial had lower proportions of enteral nutrition (0.8%) and parenteral nutrition (1.2%).^11^ Assuming that general health status of patients receiving enteral and parenteral nutrition administration is worse than that of patients not receiving this treatment, our cohort was likely to include more severely ill patients compared with clinical trials completed previously in the non-ICU setting. To further explore a potential association of this discrepancy with results in different study settings, we performed a sensitivity analysis excluding patients receiving enteral or parenteral nutrition. After matching, receiving oral nutritional support alone was associated with a 25% reduction in overall in-hospital mortality risk, which was similar to the findings by Gomes et al.^23^ In addition, our study did not assess postdischarge mortality rates, while in Gomes et al,^23^ mortality was measured up to 6 months after randomization. The shorter follow-up period in our study may also be associated with differences in survival rates.

As secondary outcomes, we assessed associations with further clinical outcomes beyond the known associations with mortality. We found reduced readmission rates in the group receiving nutritional support. Similarly, the meta-analysis by Gomes et al^23^ reported that receiving nutritional support was associated with a reduction in nonelective hospital readmissions, albeit with a large heterogeneity among the included trials. In contrast, the 2 largest randomized clinical trials of nutritional support and mortality completed to our knowledge so far, NOURISH^12^ and EFFORT,^11^ did not find significant reductions in readmission rates.

Patients in our study receiving nutritional therapy had a reduced risk of discharge to a postacute care facility, although results after accounting for competing risk showed conflicting findings. Three randomized clinical studies^24,25,26^ exploring that end point did not find a difference between intervention and control groups.

To maximize external validity, and thus provide a solid basis to generalize our findings for real-life settings, we included hospitalizations of patients with multiple morbidities, including a broad variety of comorbidities as usually seen in a real-world medical ward population. Our aim was to focus on patients in the hospital with acute illness; therefore, we did not include patients from geriatric units, who often have different characteristics (eg, more admissions to postacute care facilities).

Substantiating our second main finding of this population-based study, the EFFORT trial^11^ and the updated meta-analysis^23^ found that established or more severe malnutrition, compared with less severe malnutrition or nutritional risk only, was associated with a greater survival benefit. In our cohort, the potential survival benefits associated with a nutritional support were mostly robust across subgroups of age, reason for hospital admission, and comorbidities. However, there was no significant modification associated with the degree of malnutrition. Reductions in risk were greater in patients with lower hospital frailty risk scores and shorter hospital LOS. Whether this finding is directly associated with the nonbeneficial or even unfavorable associations of nutritional support in the critical care setting remains hypothetical.^27,28,29^

The main strengths of this study include the use of an unselected population, including patients with a variety of measurable confounding variables, accompanied by strict definitions of nutritional support (ie, high specificity) and patient outcomes. This approach is associated with reduced selection bias and increased external validity. Moreover, the study reflects highly accurate estimates at a national level, incorporating a propensity score–matched analysis of patient-centered hospital care of adult patients with malnutrition as treated in clinical routine, and thus enabling a comparative study setting. Furthermore, consistency of the results with previous findings from randomized clinical trials underlines the generalizability of these data.

### Limitations

This study has several limitations. Our data must be interpreted in the context of the study design. The diagnosis of malnutrition was based on *ICD-10* classification, and the procedure of nutritional support was based on CHOP codes, so we could not precisely stratify by the activity or severity of disease or the adequacy of nutrient replacement. As *ICD-10* codes for malnutrition are based on the NRS-2002 score,^5^ our cohort included not only patients with malnutrition, but also patients at risk for malnutrition. Nonetheless, including a broad spectrum of patients at risk for malnutrition is associated with increased external validity. In addition, a certain risk of misclassification and underreporting needs to be acknowledged, because administrative data were used and codes were provided only starting in 2013. Because of the underestimated prevalence of malnutrition, a 2017 study^30^ in Switzerland investigated the diagnostic accuracy of malnutrition codes and found a low sensitivity of 30% but a high specificity of 93.4%. In our study, attribution of the nutritional intervention was at the discretion of the treating physician, and therefore, unmeasured and unmeasurable residual confounding cannot be excluded (eg, expected benefit from nutritional support, previous nutrition therapy, or clinical or laboratory parameters). However, to approximatively address levels of frailty and disease severity, we included in our statistical model aspects of general health status, such as the Charlson Comorbidity Index, the hospital frailty risk score, and the use of palliative care.

## Conclusions

In this nationwide population-based cohort of patients with malnutrition in the hospital, receiving nutritional support as prescribed in routine care was associated with decreased in-hospital mortality compared with not receiving nutritional support. These findings were mostly consistent with those of previous large randomized clinical trials and are unlikely to be explained by unmeasured confounding.

## Notes

Supplement. **eTable 1.** Detailed Information About *ICD-10-GM* Codes of Malnutrition (Implemented in March 2013) **eTable 2.** Association of Nutritional Support With Clinical Outcomes Before and After Matching for Patients With Nutritional Support Only (Enteral and Parenteral Nutritional Interventions Excluded)
