# Supplementary material for: Evaluation of Nutritional Support and In-Hospital Mortality in Patients With Malnutrition
Source: JAMA Netw Open. 2021 Jan 20;4(1):e2033433. doi: 10.1001/jamanetworkopen.2020.33433 (PMC7818145; doi:10.1001/jamanetworkopen.2020.33433)
Supplement: Supplement. — eTable 1. Detailed Information About ICD-10-GM Codes of Malnutrition (Implemented in March 2013) eTable 2. Association of Nutritional Support With Clinical Outcomes Before and After Matching for Patients With Nutritional Support Only (Enteral and Parenteral Nutritional Interventions Excluded) [file jamanetwopen-e2033433-s001.pdf]

Supplemental Online Content

Kaegi-Braun N, Mueller M, Schuetz P, Mueller B, Kutz A. Evaluation of nutritional support and in-hospital mortality in patients with malnutrition. *JAMA Netw Open*. 2021;4(1):e2033433. doi:10.1001/jamanetworkopen.2020.33433

eTable 1. Detailed Information About *ICD-10-GM* Codes of Malnutrition (Implemented in March 2013)
eTable 2. Association of Nutritional Support With Clinical Outcomes Before and After Matching for Patients With Nutritional Support Only (Enteral and Parenteral Nutritional Interventions Excluded)

This supplemental material has been provided by the authors to give readers additional information about their work.

© 2021 Kaegi-Braun N et al. *JAMA Network Open.*

eTable 1. Detailed Information About ICD-10-GM Codes of Malnutrition (Implemented in March 2013)

Risk stratification according to NRS 2002[a)]	ICD-10-GM Codes	Definition	Criteria	Condition for coding
**Malnourished**	**E43**	**Unspecified severe protein-energy malnutrition**	NRS 2002 ≥ 5<br>and<br>one of the following criteria<br>• BMI <18.5kg/m^2<br>• Unintended weight loss of >5% in 1 month and reduced general health<br>• Reduced food intake (0-25% of the daily needs)	Combination with a CHOP code for a nutritional intervention
	**E44**	**Protein-energy malnutrition of moderate and mild degree**	E44.0 moderate:<br>NRS 2002 ≥ 4<br>and<br>one of the following criteria<br>• BMI 18.5-20.5kg/m^2<br>• Unintended weight loss of >5% in 2 months and reduced general health<br>• Reduced food intake (25-50% of the daily needs)	Combination with a CHOP code for a nutritional intervention
**At nutritional risk**			E44.1 mild:<br>NRS 2002 ≥ 3<br>and<br>one of the following criteria<br>• Unintended weight loss of >5% in 3 months and reduced general health<br>• Reduced food intake (50-75% of the daily needs)	
**Undefined**	**E46**	**Unspecified protein-energy malnutrition**	Patients who do not fulfill the criteria above or who did not have a CHOP-code for nutritional support	No conditions

Abbreviations: ICD-10-GM: International Classification of Disease, version 10, German Modification. NRS 2002: nutritional risk screening[1]. CHOP code: Swiss classification of operation code.

© 2021 Kaegi-Braun N et al. *JAMA Network Open.*

eTable 2. Association of Nutritional Support With Clinical Outcomes Before and After Matching for Patients With Nutritional Support Only (Enteral and Parenteral Nutritional Interventions Excluded)

Outcome	No nutritional support	Nutritional support	
**In-hospital all-cause mortality**			p-value
Patients, No.	36 804	64 543	
Events, No. (%)	3239 (8.8)	4932 (7.6)	
IRR (95% CI), p-value			
Unadjusted	1 [Reference]	0.74 (0.71-0.78)	<0.001
Adjusted[a]	1 [Reference]	0.73 (0.69-0.76)	<0.001
Fully adjusted[b]	1 [Reference]	0.69 (0.66-0.72)	<0.001
After propensity score matching[c]			
Patients, No.	34 027	34 027	
Events, No. (%)	2971 (8.7)	2286 (6.7)	
Analysis, IRR (95% CI), p-value	1 [Reference]	0.75 (0.71-0.80)	<0.001
**30-Day readmission rate**			
Patients, No.	33 565	59 611	
Events, No. (%)	6490 (19.3)	10 660 (17.9)	
IRR (95% CI), p-value			
Unadjusted	1 [Reference]	0.93 (0.91-0.96)	<0.001
Adjusted[a]	1 [Reference]	0.95 (0.92-0.98)	0.001
Fully adjusted[b]	1 [Reference]	0.95 (0.92-0.98)	0.003
After propensity score matching[c]			
Patients, No.	31 056	31 741	
Events, No. (%)	5884 (19.0)	5799 (18.3)	
Analysis, IRR (95% CI), p-value	1 [Reference]	0.95 (0.91-0.98)	0.003
**Discharge to post-acute care facility**			
Patients, No.	33 565	59 611	
Events, No. (%)	15 223 (45.4)	26 015 (43.6)	
OR (95% CI), p-value			
Unadjusted	1 [Reference]	0.93 (0.91-0.96)	<0.001
Adjusted[a]	1 [Reference]	0.98 (0.95-1.01)	0.120
Fully adjusted[b]	1 [Reference]	0.91 (0.88-0.94)	<0.001
After propensity score matching[c]			
Patients, No.	31 056	31 741	
Events, No. (%)	13 945 (44.9)	13 318 (42.0)	
Analysis, OR (95% CI), p-value	1 [Reference]	0.88 (0.85-0.90)	<0.001

Abbreviations: IRR: incidence rate ratio. OR: odds ratio.

[a] Adjusted for sociodemographic factors: age, sex, nationality, insurance status, month and year of admission, mode of admission, location before admission, hospital size, hospital site.

© 2021 Kaegi-Braun N et al. *JAMA Network Open.*

[b] Adjusted for sociodemographic factors and medical factors: main diagnosis, comorbidities, severity of malnutrition, total number of hospitalizations, use of palliative treatment, Charlson Comorbidity Index, hospital frailty risk score, and hospital length of stay.
[c] Sociodemographic factors and medical factors used in propensity-score matching and analyses adjusted for hospital site.

Reference:

1. Kondrup J, Rasmussen HH, Hamberg O, Stanga Z. Nutritional risk screening (NRS 2002): a new method based on an analysis of controlled clinical trials. *Clinical nutrition (Edinburgh, Scotland).* 2003;22(3):321-336.

© 2021 Kaegi-Braun N et al. *JAMA Network Open.*
